# MRI-Based Identification of an Appropriate Point of Needle Insertion for Patients with Idiopathic Carpal Tunnel Syndrome to Avoid Median Nerve Injury

**DOI:** 10.5402/2011/528147

**Published:** 2011-07-06

**Authors:** Shigeharu Uchiyama, Toshiro Itsubo, Koichi Nakamura, Hironori Murakami, Toshimitsu Momose, Hiroyuki Kato

**Affiliations:** Department of Orthopaedic Surgery, Shinshu University School of Medicine, Asahi 3-1-1, Matsumoto, Nagano 390-8621, Japan

## Abstract

To identify a safe entry point for needle insertion in patients with idiopathic carpal tunnel syndrome (CTS), cross-sectional images of the wrist MRI of 45 normal volunteers and 180 consecutive patients with idiopathic CTS were reviewed. Insertion of the needle from the five different entry points into the carpal tunnel was simulated by drawing a 1-pixel line, and the incidence of contact with the median nerve was compared. In the CTS patients, the lowest incidence was 3% when inserted at one-third of the length between the FCR and FCU tendons on the ulnar side at the level of the distal part of the distal radioulnar joint and 4% at the mid point between the palmaris longus tendona and the flexor carpi ulnaris tendon. It was greater in the advanced stage of CTS than the less severe CTS. We recommend those two entry points.

## 1. Introduction

Steroid injection into the carpal tunnel has been proved to provide temporary symptom relief in patients with carpal tunnel syndrome (CTS) [1–5]. This effect probably occurs through a decrease in the swelling of the hypertrophied synovial tissue within the carpal tunnel. This decrease in turn results in decompression of the median nerve and subsequent restoration of blood supply to the median nerve [2].

It is widely accepted that injection into the carpal tunnel should be carefully performed for the following reasons: (1) inadvertent insertion of the needle could damage the median nerve fibers and (2) glucocorticoid agents administered via intraneural injection could have toxic effects on nerve function [6–9]. Complications of carpal tunnel injection may be attributable to the following facts. Movement of the median nerve in patients with CTS is compromised as compared to that in normal individuals [10]. Thus, the chances of the nerve escaping needle piercing by sliding toward the ulnar or radial directions are low. Furthermore, proximal swelling of the median nerve at the entrance of the carpal tunnel is remarkable, especially in the advanced stages of the condition [11].

Although many injection sites have been identified in previous studies [2, 12–20], comparative studies have not been performed in patients with different stages of CTS. Furthermore, regardless of the entry portal, contact with the median nerve, even if unusual, is possible; in such a case, the needle should be retracted subcutaneously and either its direction changed or a new entry point sought. An appropriate insertion point would be the one through which there is minimum likelihood for contact with the median nerve and one that allows introduction of the needle from a wide range of angles. In this study, we used magnetic resonance imaging (MRI) to simulate the path of a needle inserted adjacent to or inside the carpal tunnel by using 5 different insertion points. We aimed to determine the entry point at which the possibility for contact with the median nerve was minimal and the introduction of the needle from the widest range of angles was possible, without any contact with the surrounding neurovascular bundles. 

## 2. Materials and Methods

**Figure 1:**
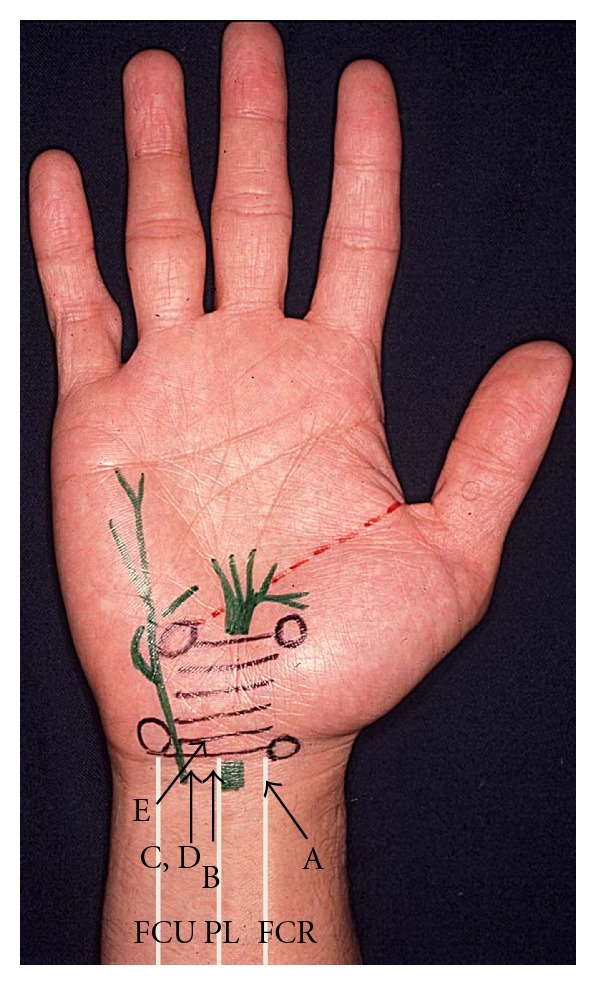
Five entry points of injection. A: through the flexor carpi radialis (FCR) tendon radially tilted at 45 degrees, B: close to the ulnar side of the palmaris longus tendon (PL) tendon, C: at the midpoint between the PL and the flexor carpi ulnaris (FCU) tendons, D: at one-third of the length between the FCR and FCU tendons on the ulnar side, and E: in line with the fourth ray at the pisiform level. Entry points of A, B, C, and D are at the level of the distal part of the distal radioulnar joint.

We retrospectively reviewed the cases of 45 normal volunteers without symptoms of CTS or any delay in median nerve conduction across the wrist and 180 consecutive patients with idiopathic CTS. MRI data of some of these volunteers have been reported elsewhere [11]. The mean (standard deviation) age of the normal volunteers was 48 (11) years, and that of the CTS patients was 60 (13) years. The CTS patients included 14 men, whereas all the volunteers comprised only women. Among the patients, 58 were deemed to have moderate CTS with motor distal latency (MDL) greater than 4.4 ms and sensory conduction velocity less than 44 m/s. Further, 74 patients were deemed to have severe CTS with delayed MDL and no sensory nerve action potential response. Finally, 48 patients were deemed to have extremely severe CTS with neither compound muscle action potential nor sensory nerve action potential in response to electrical stimuli delivered to the wrist. All the patients with extremely severe CTS exhibited severe thenar muscle atrophy with loss of the normal volar prominence of the muscle belly. MRI of the right wrist in the case of all the volunteers and of both the wrists in that of the patients was performed. The detailed MRI protocol has been previously described [11]. Each participant was asked to remain in a prone position with his/her arm extended over the head, the forearm and wrist in a neutral position, and the fingers extended. As per a recent protocol, the wrist is maintained in a different position. Further, the patients are asked to remain in a supine position with their forearms in a neutral position and their wrists slightly extended. Cross-sections at the level of the distal part of the distal radioulnar joint were used for entry points A, B, C, and D; one at the level of the pisiform was used for entry point E (Figure 1).

**Figure 2:**
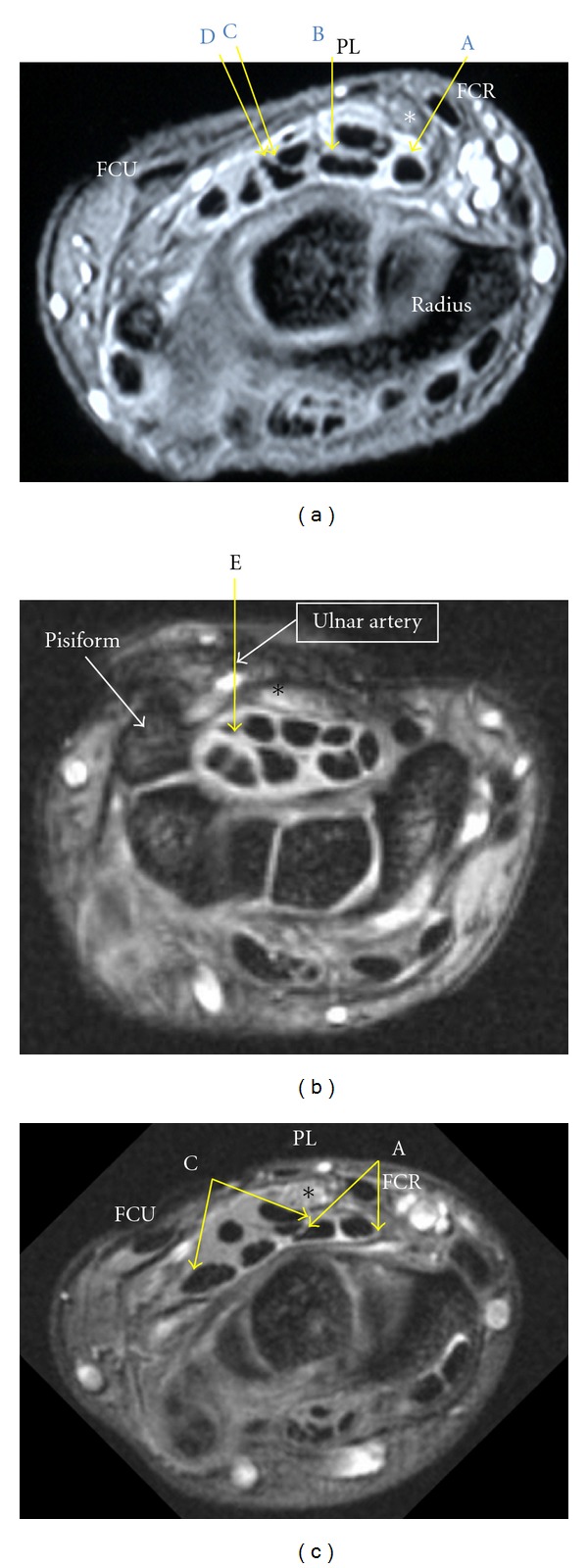
(a) At the level of the distal part of the distal radioulnar joint. Entry points A, B, C, and D are shown. Insertion from any of these points does not result in contact between the needle and the median nerve. *Median nerve. (b) At the level of the distal wrist crease. Entry point E, which is in line with the fourth ray, is shown. Insertion from this results in contact between the needle and the ulnar artery. (c) At the level of the distal part of the distal radioulnar joint. The range of angles provided by points A and C, insertion at which prevents contact between the needle and the neurovascular structures, is shown.

Insertion of the needle from the following entry points into the carpal tunnel was simulated by drawing a 1-pixel line: A, through the flexor carpi radialis (FCR) tendon radially tilted at 45 degrees in the horizontal plane [18], B, just ulnar to the palmaris longus (PL) tendon [2, 12, 14] and directed perpendicular to the horizontal plane C, at the midpoint between the ulnar edge of the PL tendon and the radial edge of the flexor carpi ulnaris (FCU) tendon [15] and perpendicular to the horizontal plane D, at one-third of the length between the ulnar edge of the FCR tendon and the radial edge of the FCU tendon from the FCU tendon and perpendicular to the horizontal plane E, at the level of the distal wrist crease in line with the fourth ray [16] and directed perpendicular to the horizontal plane (Figure 2). 

The incidence of contact with the median nerve was determined by drawing a line through each entry portal, and this incidence was compared between the patients with different stages of CTS and the normal volunteers by using Fisher's exact test. For the CTS patients, we determined the range of the angle at points from which the needle could be inserted into the carpal tunnel along the axial plane without damaging the neurovascular structures such as the median nerve, ulnar nerve, radial artery, or ulnar artery (Figure 2). We compared this range among the insertion points and also among the patients with different stages of CTS by using paired or unpaired *t*-test. The data for entry point E were not available for normal individuals and were available for only 71 CTS patients who underwent MRI while in a supine position. Statistical significance was determined when the *P* value was less than 0.05.

The ethical committee of our institution approved the study protocol.

## 3. Results

The incidence of contact with the median nerve when the needle was inserted at any of the 5 different entry points in the normal subjects and CTS patients was shown in Table 1.

 In the CTS patients, this incidence was 8%, 4%, 3%, and 11% when the needle was inserted from entry points A, C, D, and E, respectively, but 70% when inserted through B. Further, the likelihood of the needle making contact with the median nerve through entry point B was greater in the CTS patients than in the volunteers (*P* < 0.0001). In the case of the CTS patients, the chances of contact with the median nerve were higher when the needle was inserted from point D than from point A (*P* = 0.036). The chances of contact with the median nerve when the needle was inserted through C were higher in patients with severe CTS than in those with moderate CTS (*P* = 0.0209) (Table 2). 

When the needle was inserted through point E, it made contact with the ulnar artery in 12 of 71 patients (20%).

Compared to point A, points C and D provided a wider range of angles over which the needle did not make contact with the neurovascular structures regardless of the severity of the CTS (*P* < 0.006) (Table 3).

The range of angles over which the needle did not make contact with the neurovascular structures was greater in patients with extremely severe CTS than in those with moderate or severe CTS (*P* < 0.0001) (Table 3).

**Figure 3:**
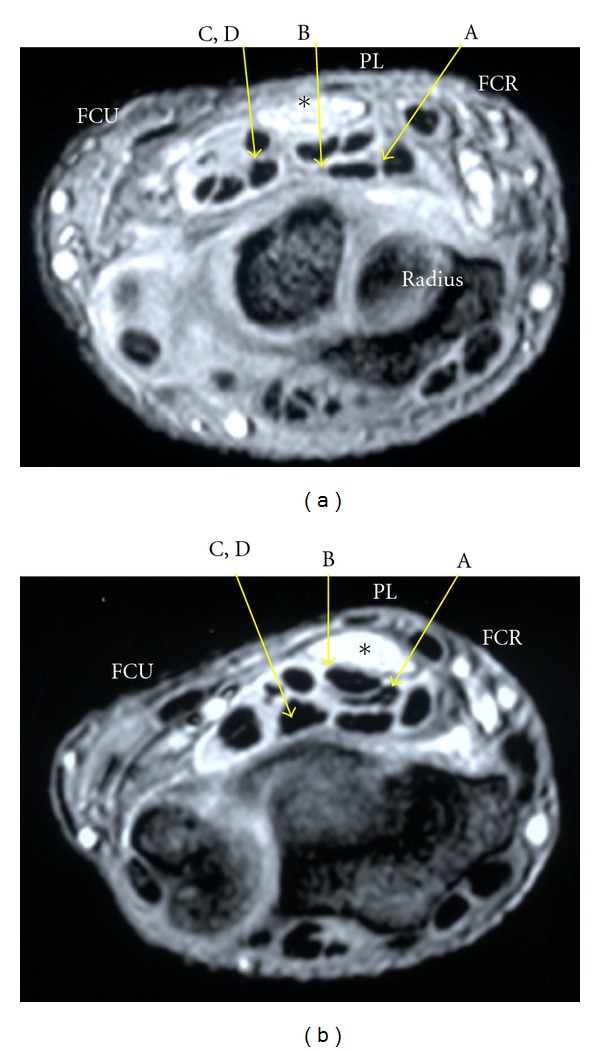
(a) Wrist of a patient with severe CTS at the level of the distal part of the distal radioulnar joint. The median nerve is enlarged and can be seen as a high-intensity area on the MRI. Insertion from the entry point close to the ulnar side of the PL results in contact between the needle and the nerve. Insertion from C and D leaves a narrow margin between the needle and the nerve. *Median nerve. (b) Wrist of a patient with extremely severe CTS. At the level of the distal part of the distal radioulnar joint, the median nerve is enlarged and can be seen as a high-intensity area on the MRI. Insertion from the entry point close to the ulnar side of the PL and FCR results in contact between the needle and the nerve. *Median nerve.

Representative results are shown in Figure 3.

## 4. Discussion

This study revealed that when a needle was inserted through an entry point located at the midpoint between the PL and FCU tendons or at one-third of the length between the FCR and FCU tendons on the ulnar side, in the absence of the PL tendon, was less likely to make contact with the median nerve than when it was inserted through other entry portals at the distal part of the distal radioulnar joint level. Furthermore, the former entry points allowed a wider range of angles over which the needle could be inserted without damaging important structures. These entry points can be easily identified in daily clinical practice. In addition, the likelihood of contact with the median nerve when the needle was inserted through a point in line with the fourth ray at the pisiform level was similar to that observed in the case of the entry point located at the midpoint between the PL and FCU tendons or that at one-third of the length between the FCR and FCU tendons on the ulnar side; however, needle insertion at this point can damage the ulnar artery inside Guyon's canal. In either case, the possibility of the needle making contact with the median nerve does exist.

To avoid injury of the median nerve, insertion at a more proximal site, for example, 3-4 cm proximal to the wrist crease, can be an alternative [17, 21]. Minamikawa et al. injected 2 mL of a dye at a point 3 cm proximal to the distal flexion crease of the wrist in freshly frozen cadaveric hands and found that a sufficient amount of the dye diffused into the carpal tunnel [22]. However, it is unclear whether this approach can be applied in the case of living patients with hypertrophied synovial tissue, which can preclude distal diffusion of the injected agent.

Some researchers recommend needle insertion through the FCR tendon because the chances of damage to the median nerve are minimal through this point [18]. However, our study revealed that a needle inserted through the FCR tendon can pierce the median nerve and that the range of angles over which this insertion is safe is not wide.

Recently, Smith et al. introduced a sonography-guided ulnar approach for needle insertion [23]. This approach enables needle insertion without any contact with the median nerve; however, employing sonography in a busy outpatient unit may not be feasible in most institutions.

If a patient experiences radiating pain during needle insertion, the needle should be retracted. In this case, an alternative path of needle insertion should be carefully identified, because the range of angles for a safe insertion is narrow even at the midpoint between the PL and FCU tendons or at one-third of the length between the FCR and FCU tendons on the ulnar side. Reinsertion through a different entry point, for example, through the FCR or more proximal insertion may be an alternative.

The present study is the first wherein the chances of a contact with the median nerve were determined using simulated needle insertion and wherein the different outcomes with different points of insertion were statistically compared. It should be noted that because the median nerve of the wrist may not be swollen in cadavers, we used images of CTS patients, which probably better reflected the cases in clinical settings [20, 24]. 

A limitation of this study is that the position of the forearm or wrist may have affected the results to some extent because of variations in the position of the median nerve in relation to superficially located tendons such as the PL, FCR, or FCU. In this study, we performed a two-dimensional analysis to assess the insertion angle. The chances of contact with the median nerve when the needle is inserted at an angle directed toward the sagittal and axial planes, as recommended by most previous investigators, remain unknown. However, we believe that the insertion of the needle along the sagittal or axial plane may not significantly affect the outcome, because the median nerve is located in superficial position around the level of the distal part of the distal radioulnar joint. The effect of the depth of needle insertion was not considered in this study.

## Figures and Tables

**Table 1:** Rate of contact to the median nerve through 5 different insertion points in normal subjects and CTS patients.

	A: FCR	B: PL	C: PL&FCU	D: FCR&FCU	E: Distal crease
Normal *n* = 45 (no PL: 7)	7 (16%)	*12 (27%)	0	1 (2%)	NA
CTS *n* = 180 (7)	**15 (8%)	*121 (70%)	7 (4%)	**5 (3%)	8/71 (11%)

*P < 0.0001, **P = 0.036.

FCR: through flexor carpi radialis tendon. PL: just ulnar to PL tendon. PL&FCU: 1/2 between PL tendon and FCU tendon.

FCR&FCU: ulnar 1/3 between FCU tendon and FCR tendon. Distal crease: distal wrist crease level in line with the 4th ray.

NA: data not available. Seventy-one patients are available for analysis in entry point E.

**Table 2:** Rate of contact to the median nerve through 5 different insertion points in 3 stages of CTS patients.

Stage		A: FCR	B: PL	C: PL&FCU	D: FCR&FCU	E: Distal crease
Moderate	58 (no PL: 2)	5 (8.6%)	37 (66.1%)	*0	**0	1 (*n* = 23, 4.3%)
Severe	74 (2)	5 (6.8%)	47 (65.3%)	2 (2.8%)	1 (1.4%)	1 (*n* = 24, 4.2%)
Extremely severe	48(3)	5 (10.4%)	37 (82.2%)	*5 (11.1%)	**4 (8.3%)	6 (*n* = 24, 25%)

*P = 0.014, **P = 0.039.

**Table 3:** Range of angle (degrees) without contacting the median nerve, ulnar nerve, radial artery, or ulnar artery through 4 different insertion points.

Stages	A: FCR	C: PL&FCU	D: FCR&FCU	E: Distal crease
Moderate	47 ± 14	77 ± 19	78 ± 22	18 ± 14
Severe	47 ± 16	74 ± 18	75 ± 19	26 ± 16
Extremely severe	49 ± 16	59 ± 22	59 ± 23	22 ± 13

Insertion point E has smaller angle than the other sites in any severities of the stage: *P* < 0.0001.

Insertion point A has smaller angle than C or D in any severities of the stage: moderate and severe, *P* < 0.0001 extremely severe, *P* < 0.006.

In insertion point C and D, extremely severe stage has smaller angle than the other stages. *P* < 0.0001.

## References

[1] Goodman HV, Foster JB (1962). Effect of local corticosteroid injection on median nerve conduction in carpal tunnel syndrome. *Annals of Physical Medicine*.

[2] Phalen GS (1966). The carpal-tunnel syndrome. Seventeen years’ experience in diagnosis and treatment of six hundred fifty-four hands. *Journal of Bone and Joint Surgery*.

[3] Wood MR (1980). Hydrocortisone injections for carpal tunnel syndrome. *Hand*.

[4] Marshall S, Tardif G, Ashworth N (2007). Local corticosteroid injection for carpal tunnel syndrome. *Cochrane Database of Systematic Reviews*.

[5] Keith MW, Masear V, Amadio PC (2009). Treatment of carpal tunnel syndrome. *The Journal of the American Academy of Orthopaedic Surgeons*.

[6] Mackinnon SE, Hudson AR, Gentili F (1982). Peripheral nerve injection injury with steroid agents. *Plastic and Reconstructive Surgery*.

[7] McConnell JR, Bush DC (1990). Intraneural steroid injection as a complication in the management of carpal tunnel syndrome: a report of three cases. *Clinical Orthopaedics and Related Research*.

[8] Kasten SJ, Louis DS (1996). Carpal tunnel syndrome: a case of median nerve injection injury and a safe and effective method for injecting the carpal tunnel. *Journal of Family Practice*.

[9] Tavares SP, Giddins GEB (1996). Nerve injury following steroid injection for carpal tunnel syndrome. A report of two cases. *Journal of Hand Surgery*.

[10] Nakamichi K, Tachibana S (1995). Restricted motion of the median nerve in carpal tunnel syndrome. *Journal of Hand Surgery*.

[11] Uchiyama S, Itsubo T, Yasutomi T, Nakagawa H, Kamimura M, Kato H (2005). Quantitative MRI of the wrist and nerve conduction studies in patients with idiopathic carpal tunnel syndrome. *Journal of Neurology, Neurosurgery and Psychiatry*.

[12] Foster JB (1960). Hydrocortisone and the carpal-tunnel syndrome. *Lancet*.

[13] Gelberman RH, Aronson D, Weisman MH (1980). Carpal-tunnel syndrome. Results of a prospective trial of steroid injection and splinting. *Journal of Bone and Joint Surgery*.

[14] Green DP (1984). Diagnostic and therapeutic value of carpal tunnel injection. *Journal of Hand Surgery*.

[15] Frederick HA, Carter PR, Littler JW (1993). Injection injuries to the median and ulnar nerves at the wrist. *Journal of Hand Surgery*.

[16] Kay NR, Marshall PD (1992). A safe, reliable method of carpal tunnel injection. *Journal of Hand Surgery*.

[17] Dammers JW, Veering MM, Vermeulen M (1999). Injection with methylprednisolone proximal to the carpal tunnel: randomised double blind trial. *British Medical Journal*.

[18] Racasan O, Dubert T (2005). The safest location for steroid injection in the treatment of carpal tunnel syndrome. *Journal of Hand Surgery*.

[19] Habib GS, Badarny S, Rawashdeh H (2006). A novel approach of local corticosteroid injection for the treatment of carpal tunnel syndrome. *Clinical Rheumatology*.

[20] MacLennan A, Schimizzi A, Meier KM, Barron OA, Catalano L, Glickel S (2009). Comparison of needle position proximity to the median nerve in 2 carpal tunnel injection methods: a cadaveric study. *Journal of Hand Surgery*.

[21] Sevim S, Dogu O, Camdeviren H (2004). Long-term effectiveness of steroid injections and splinting in mild and moderate carpal tunnel syndrome. *Neurological Sciences*.

[22] Minamikawa Y, Peimer CA, Kambe K, Wheeler DR, Sherwin FS (1992). Tenosynovial injection for carpal tunnel syndrome. *Journal of Hand Surgery*.

[23] Smith J, Wisniewski SJ, Finnoff JT, Payne JM (2008). Sonographically guided carpal tunnel injections: the ulnar approach. *Journal of Ultrasound in Medicine*.

[24] Ozturk K, Esenyel CZ, Sonmez M, Esenyel M, Kahraman S, Senel B (2008). Comparison of carpal tunnel injection techniques: a cadaver study. *Scandinavian Journal of Plastic and Reconstructive Surgery and Hand Surgery*.

